# Supplementary material for: Thermal metamaterials for enhanced non-Fourier heat transport
Source: NPJ Metamater. 2025 Nov 18;1(1):7. doi: 10.1038/s44455-025-00008-3 (PMC12637368; doi:10.1038/s44455-025-00008-3)
Supplement: Supplementary file 1 — Supplementary Information [file 44455_2025_8_MOESM1_ESM.pdf]

Supplementary Material
Thermal Metamaterials for Enhanced Non-Fourier Heat Transport

Harry Mclean,[1] Francis Huw Davies,[1] Ned Thaddeus Taylor,[1] and Steven Paul Hepplestone[1]

[1]*Department of Physics and Astronomy, University of Exeter, Stocker Road, Exeter, EX4 4QL, United Kingdom*

(Dated: August 26, 2025)

CONTENTS

S1. Methodology S2
S1.1. Full Cattaneo Derivation from Perturbation Using the Boltzmann Transport Equation S2
S1.1.1. Microscopic model S2
S1.1.2. Macro-scale correspondence S5
S1.1.3. Inclusion of a power term S5
S1.2. Resolving negative temperature failings of the Cattaneo approach S7
S1.3. Wave-like assumptions and consequences S9
S1.4. One-dimensional analytical solution of the macroscopic Cattaneo equation S10
S2. Modelling S12
S3. Supporting Results S13
References S30

S1. METHODOLOGY

S1.1. Full Cattaneo Derivation from Perturbation Using the Boltzmann Transport Equation

In this section, we derive the Cattaneo equation starting from the Boltzmann Transport Equation (BTE) under the influence of a perturbation. This derivation captures the finite speed of thermal propagation and introduces phonon relaxation effects.

S1.1.1. Microscopic model

The BTE describes the evolution of the distribution function f, which depends on momentum, position, and time. Our approach follows previous works by authors such as Klemens [1], Callaway [2], and Srivastava [3]. Thus, we start with a non-equilibrium distribution function:

$$f = f\left(\mathbf{k}, s, \mathbf{r}, t\right), \tag{S1}$$

where f is a function of crystal momentum $\mathbf{k}$, band mode s, position $\mathbf{r}$, and time t. To proceed, we apply the chain rule to expand the total time derivative of f:

$$\frac{df}{dt} = \frac{\partial f}{\partial \mathbf{k}} \cdot \frac{d\mathbf{k}}{dt} + \frac{\partial f}{\partial s} \cdot \frac{ds}{dt} + \frac{\partial f}{\partial \mathbf{r}} \cdot \frac{d\mathbf{r}}{dt} + \frac{\partial f}{\partial t}. \tag{S2}$$

Here, the left-hand side represents the scattering/collision rate, while the right-hand side consists of local diffusion and local accumulation terms. This is the standard approach for the BTE [3]. By introducing the relations $\frac{d\mathbf{k}}{dt} = F$ (where F is the force acting on the particles) and $\frac{d\mathbf{r}}{dt} = \upsilon$ (the velocity of particles), we can rewrite the equation as:

$$\frac{df}{dt} = \frac{\partial f}{\partial \mathbf{k}} F + \frac{\partial f}{\partial s} \frac{ds}{dt} + \frac{\partial f}{\partial \mathbf{r}} \upsilon + \frac{\partial f}{\partial t}. \tag{S3}$$

In phonon transport, the force F is generally set to zero because phonons are massless particles. Additionally, we assume that band modes s are time-independent. Therefore, Eq. (S3) simplifies to:

$$\frac{df_{\mathbf{k}s}}{dt} = \frac{\partial f_{\mathbf{k}s}}{\partial \mathbf{r}} \cdot \upsilon_{\mathbf{k}s} + \frac{\partial f_{\mathbf{k}s}}{\partial t}. \tag{S4}$$

where $\frac{df_{\mathbf{k}s}}{dt}$ is the total rate of change of the distribution with time, which can be shown to be the scattering term. This can be described as

$$\left.\frac{\partial f_{\mathbf{k}s}}{\partial t}\right|_{scattering} = \left.\frac{\partial f_{\mathbf{k}s}}{\partial t}\right|_{diff} + \left.\frac{\partial f_{\mathbf{k}s}}{\partial t}\right|_{local}. \tag{S5}$$

The distribution $f_{\mathbf{k}s}$ can be expressed as the non-equilibrium distribution, f_0 is the equilibrium distribution, and the time between the change in these distributions is τ.

Under the relaxation time approximation, the scattering rate is given by:

$$\left.\frac{\partial f_{\mathbf{k}s}}{\partial t}\right|_{scattering} = -\frac{f_{\mathbf{k}s} - f^0_{\mathbf{k}s}}{\tau_{\mathbf{k}s}} \tag{S6}$$

Substituting this into Eq. (S5), we obtain:

$$-\frac{f_{\mathbf{k}s} - f^0_{\mathbf{k}s}}{\tau_{\mathbf{k}s}} = \frac{\partial f_{\mathbf{k}s}}{\partial \mathbf{r}} \upsilon_{\mathbf{k}s} + \frac{\partial f_{\mathbf{k}s}}{\partial t}. \tag{S7}$$

This condition is similar to most relaxation time approaches and implies that the non-equilibrium distribution is reached within a relaxation time τ.

Multiplying by $\frac{-1}{V}\hbar\omega_{\mathbf{k}s}\upsilon_{\mathbf{k}s}\tau_{\mathbf{k}s}$, we get

$$\frac{1}{V}\hbar\omega_{\mathbf{k}s}\upsilon_{\mathbf{k}s}(f_{\mathbf{k}s} - f^0_{\mathbf{k}s}) = -\frac{1}{V}\hbar\omega_{\mathbf{k}s}\tau_{\mathbf{k}s}\upsilon_{\mathbf{k}s} \otimes \upsilon_{\mathbf{k}s}\frac{\partial f_{\mathbf{k}s}}{\partial \mathbf{r}} - \frac{1}{V}\frac{\partial(\hbar\omega_{\mathbf{k}s}\upsilon_{\mathbf{k}s}\tau_{\mathbf{k}s}f_{\mathbf{k}s})}{\partial t}. \tag{S8}$$

Note we use the outer product $\vec{v}_{\mathbf{k}s} \times (\vec{v}_{\mathbf{k}s} \cdot \vec{\nabla} f_{\mathbf{k}s}) = (\upsilon_{\mathbf{k}s} \otimes \upsilon_{\mathbf{k}s})\nabla f_{\mathbf{k}s}$ to represent the vector combination of group velocity and the gradient function. This is convenient as it allows us to express thermal conductivity as a tensor.

We can now make a substitution of,

$$\frac{\partial f_{\mathbf{k}s}}{\partial \mathbf{r}} = \frac{\partial f_{\mathbf{k}s}}{\partial T}\frac{\partial T}{\partial \mathbf{r}} = \frac{\partial f_{\mathbf{k}s}}{\partial T}\nabla T, \tag{S9}$$

into Eq. (S8),

$$\frac{1}{V}\hbar\omega_{\mathbf{k}s}\upsilon_{\mathbf{k}s}(f_{\mathbf{k}s} - f^0_{\mathbf{k}s}) = -\frac{1}{V}\hbar\omega_{\mathbf{k}s}\tau_{\mathbf{k}s}\upsilon_{\mathbf{k}s} \otimes \upsilon_{\mathbf{k}s}\frac{\partial f_{\mathbf{k}s}}{\partial T}\nabla T - \frac{1}{V}\frac{\partial}{\partial t}\tau_{\mathbf{k}s}\hbar\omega_{\mathbf{k}s}\upsilon_{\mathbf{k}s}f_{\mathbf{k}s}. \tag{S10}$$

Taking the summation (over all $\mathbf{k}s$) of Eq. (S10), we obtain:

$$\frac{1}{V}\sum_{\mathbf{k}s}\hbar\omega_{\mathbf{k}s}\upsilon_{\mathbf{k}s}\left(f_{\mathbf{k}s}-f^0_{\mathbf{k}s}\right)=-\frac{1}{V}\sum_{\mathbf{k}s}\hbar\omega_{\mathbf{k}s}\tau_{\mathbf{k}s}\upsilon_{\mathbf{k}s}\otimes\upsilon_{\mathbf{k}s}\frac{\partial f_{\mathbf{k}s}}{\partial T}\nabla T-\frac{1}{V}\frac{\partial}{\partial t}\sum_{\mathbf{k}s}\tau_{\mathbf{k}s}\hbar\omega_{\mathbf{k}s}\upsilon_{\mathbf{k}s}f_{\mathbf{k}s}. \tag{S11}$$

Here (As with the Klemens approach [1] etc.) we say that the contribution to flux from the equilibrium distribution must be zero ($0=\sum_{\mathbf{k}s}\hbar\omega_{\mathbf{k}s}\upsilon_{\mathbf{k}s}f^0_{\mathbf{k}s}$). We can now use the known expressions for heat flux

$$q=\frac{1}{V}\sum_{\mathbf{k}s}\hbar\omega_{\mathbf{k}s}\upsilon_{\mathbf{k}s}f_{\mathbf{k}s}, \tag{S12}$$

and the steady state (non-varying in time) thermal conductivity (derived normally via the Klemens approach, etc).,

$$\kappa=\frac{1}{V}\sum_{\mathbf{k}s}\hbar\omega_{\mathbf{k}s}\tau_{\mathbf{k}s}\upsilon_{\mathbf{k}s}\otimes\upsilon_{\mathbf{k}s}\frac{\partial f_{\mathbf{k}s}}{\partial T}, \tag{S13}$$

and substitute them into Eq. (S11)) resulting in,

$$q=-\tau\frac{\partial q}{\partial t}-\kappa\nabla T, \tag{S14}$$

where τ is an averaged relaxation time (such that $\tau_{\mathbf{k}s}$ can be approximated as $\tau_{\mathbf{k}s}=\tau=$ constant), and q^0 is the equilibrium heat flux, which must of course be 0, leading to the Cattaneo equation:

$$-\kappa\nabla T=q+\tau\frac{\partial q}{\partial t}. \tag{S15}$$

S1.1.2. Macro-scale correspondence

We can take the divergence of Eq. (S15),

$$\nabla \cdot (\kappa \nabla T) = -\nabla \cdot q - \tau \frac{\partial \nabla \cdot q}{\partial t}. \tag{S16}$$

We can use the correspondence principle to identify terms with their macro-scale quantities. To begin with, we note the continuity equation,

$$\rho \frac{\partial U}{\partial t} = -\nabla \cdot q, \tag{S17}$$

and the internal energy

$$\frac{\partial U}{\partial t} = C_V \frac{\partial T}{\partial t}. \tag{S18}$$

Here ρ is the density and C_V is the specific heat capacity. We substitute Eqs. (S17) and (S18) into the divergence of Eq. (S15) and thus retrieve the traditional Cattaneo equation for heat flow:

$$\nabla \cdot (\kappa \nabla T) = \rho C_V \frac{\partial T}{\partial t} + \tau \rho C_V \frac{\partial^2 T}{\partial t^2}. \tag{S19}$$

S1.1.3. Inclusion of a power term

In the previous treatment, we ignored the effect of including the power source. Substitution of a power term into the continuity equation, Eq. (S17) and then following on with the substitution into the heat flux equation results in a modified Cattaneo equation, Eq. (S19),

$$\rho C_V \frac{dT}{dt} + \rho C_V \tau \frac{d^2 T}{dt^2} - (P + \tau \frac{dP}{dt}) = \nabla \cdot (\kappa \nabla T). \tag{S20}$$

where P is the power density (power per unit volume). This approach yields a heat flow equation that takes into account time-dependent power contributions, thereby enhancing the symmetry between the energy stored and the added energy. It is also known as the 'pseudo source' term and is a consequence of the inertia in the heat flux [4].

The standard heat flow equation with an additional power time dependence only affects time-dependent (oscillatory) heating cases. It is clear to see the significance of this term

when a high frequency ($\geq \tau$) AC power term is used. It is clear that for any constant heating sources, any delta-like transient effects will cause the pseudo-source term to dominate, which requires careful consideration when used.

S1.2. Resolving negative temperature failings of the Cattaneo approach

A common criticism of the Cattaneo and similar hyperbolic heat transport models is their prediction of transient temperature oscillations below thermal equilibrium, which can imply physically unrealistic negative temperatures. At sufficiently high temperatures, where fluctuations occur well above absolute zero and temperature-dependent material properties remain largely constant, the wave-like behaviour described by the Cattaneo equation becomes effectively temperature-independent, governed primarily by the second-order time derivative of temperature. However, careful attention must be paid to low-temperature regimes, where material properties exhibit strong temperature dependence, and absolute zero may lie within the range of these hyperbolic temperature fluctuations.

At low temperatures, interface/boundary scattering dominates phonon interactions, typically represented by a constant relaxation time, τ. Under these conditions, the thermal energy corresponds primarily to a single occupied vibrational mode ($k_{\mathrm{B}}T = \hbar\omega$). Thus, by the principle of energy conservation, the scattered mode must be the same as the incident mode. Consequently, the phonon occupation number does not change over time, implying that $f(0) - f(t) = 0$. According to equation Eq. (S6), this results in the scattering term, M/τ, approaching zero as $T \to 0$. At the lowest temperature, only the fundamental vibrational mode remains active, precluding any inelastic scattering processes and restricting interactions solely to elastic two-particle scattering events.

For boundary/interface scattering at low temperatures, where τ is considered constant, we have a single vibration mode filled ($k_{\mathrm{B}}T = \hbar\omega$) and thus, by energy conservation, the scattered mode must be the same as the incident mode. Hence, the rate of change of f with time must be zero as no new states are created or lost (i.e. $f = f^0$), so $f(0) - f(t) = 0$. Therefore, Eq. (S6) shows that M/τ tends to zero as T→0. Thus, the lowest energy mode cannot split (as it is the lowest energy mode) and at the lowest temperature is the singular mode of vibration, so it can not interfere with another state, i.e. only elastic 2-particle scattering is allowed.

At the macro-scale, the appearance of negative temperatures requires constant heat capacity and conductivity as the temperature approaches zero. However, as the temperature tends to zero, so does the heat capacity [5], and thus, no negative temperature is possible if

one considers any real physical material with temperature-dependent properties. Our particle equation (Eq. 2 in the main manuscript) obeys this, and it is only when the macro-scale simplifications (i.e. switch to constant C_v, κ, etc.) are applied that the risk of negative absolute temperatures occurs.

S1.3. Wave-like assumptions and consequences

To emphasise that wave-like behaviour in Fourier is an invalid solution, we consider here the implications of solving the Cattaneo/Fourier equation with a temperature plane wave of the form $T = T_o e^{-(i(\mathbf{k}.\mathbf{r}-\omega t))}$.

Starting from the Cattaneo-like equation in the form (all material parameters are 1),

$$p\frac{\partial^2 T}{\partial t^2} + \frac{\partial T}{\partial t} = \nabla^2 T, \tag{S21}$$

where t is time, T is temperature, and p is our Cattaneo parameter. When $p = 0$, this equation reduces to the Fourier equation, and $p = 1$ is the Cattaneo form. We can substitute a 1D plane wave function of T of the form

$$T = T_0 e^{-i(kx-\omega t)}, \tag{S22}$$

where k is the wave number and ω is the angular frequency. Substituting Eq. (S22) into Eq. (S21), provides a solution of the form

$$p\omega^2 - i\omega - k^2 = 0. \tag{S23}$$

This, in turn, gives valid solutions for ω of the form,

$$\omega = \frac{i \pm \sqrt{-1 + 4pk^2}}{2p}. \tag{S24}$$

When $p = 1$, we have a valid solution for a decaying plane wave. However, when observing the limit as p tends to 0,

$$\lim_{p\to 0} \omega = \frac{i \pm i}{2p}, \omega = 0; \frac{1}{p} \tag{S25}$$

. The only valid solution is 0, implying that Fourier's law does not support wave-like temperature dependence.

We can also solve direction Eq. (S23), in the case $p = 0$, for the same temperature wave,

$$-i\omega = k^2, \tag{S26}$$

which again suggests,

$$\omega = -ik^2. \tag{S27}$$

The imaginary dispersion relation is non-propagatory and will result in a decay-dominated strictly diffusive heat transfer. This is not consistent with a picture of heat carried by phonon waves.

S1.4. One-dimensional analytical solution of the macroscopic Cattaneo equation

Although our model does not enforce wave-like temperature propagation, as our model uses a finite difference method, we can analyse the macroscopic equation using plane waves for analytical purpose.

We model one–dimensional heat transport, in a rod of length L, With a hot side H_h and a cold side H_c, using the Cattaneo equation in the following one-dimensional form

$$\alpha \frac{d^2 T}{dx^2} = \frac{\partial T}{\partial t} + \tau_{\text{Cat}} \frac{\partial^2 T}{\partial t^2}. \qquad 0 < x < L,\ t > 0, \tag{S28}$$

where α is the thermal diffusivity and τ the relaxation time. The boundary and initial conditions are

$$T(0,t) = H_h, \quad T(L,t) = H_c, \quad T(0 < x < L, 0) = H_c, \qquad T_t(x,0) = 0. \tag{S29}$$

The asymptotic solution (steady state) is readily known can can be used to reduce the problem to the homogeneous condition. Defining $\Delta H = H_h - H_c$, the time asymptotic profile is,

$$T_\infty(x) = H_c + \Delta H \left(1 - \frac{x}{L}\right). \tag{S30}$$

We may now write, $T(x,t) = T_\infty(x) + \theta(x,t)$ and because T_∞ is stationary, θ obeys the homogeneous form of (S1) with

$$\theta(0,t) = \theta(L,t) = 0, \tag{S31}$$

$$\theta(x,0) = -\Delta H \left(1 - x/L\right). \tag{S32}$$

Separating variables in the usual way yields a Fourier series solution in the spacial component, and the temporal function is comprised of forwards and backwards propagating waves subject to a uniform decay factor. Hence the full solution is

$$\boxed{T(x,t) = T_\infty(x) - \frac{2\Delta H}{\pi} e^{-t/2\tau} \sum_{n=1}^{\infty} \frac{\sin(k_n x)}{n} \left[\cos(\zeta_n t) + \frac{\sin(\zeta_n t)}{2\tau\zeta_n}\right]}. \tag{S33}$$

where the modal frequency is

$$\zeta_n = \sqrt{\frac{\alpha k_n^2}{\tau} - \frac{1}{4\tau^2}} \tag{S34}$$

and the wavenumber is

$$k_n = \frac{n\pi}{L}. \tag{S35}$$

We can analyse the solution given by Eq. S33 to determine the limits of wave-like behaviour. The modular frequency ζ_n gives the breakdown condition depending on whether it is real or imaginary. When ζ_n is imaginary the heat flow is purely diffusive. However, $\zeta + n$ is real, the heat flow is a combination of diffusive and wave-like propagation. The uniform decay term $e^{-t/2\tau}$ is clearly dominant and immediately demonstrates that the decay times are comparable with the relaxation time (as expected).

An effective method to identify a wave-like regime is to define a critical length $\mathcal{L}_c = 4\pi\sqrt{\alpha\tau}$ below which $L < \mathcal{L}_c$ wave-like behaviour will be seen. Above this critical length $L > \mathcal{L}_c$, all heat transfer will appear diffusive. As this series consists of n modes, the wave-like term will always appear (critical to resolving the instantaneous transfer dilemma) but will not dominate.

S2. MODELLING

We have implemented a model of Eq. (S19) using implicit finite difference. The full implementation can be found at the publicly available GitHub page `https://github.com/ExeQuantCode/HeatFlow`, and via figshare (doi:10.6084/m9.figshare.27979781). In our modelling (in the systems shown in Fig. S1), we focus on the flow of heat in solid materials; thus, radiative and convective effects have been omitted. The thermal conductivity between regions of different materials is calculated as a harmonic average, a standard method in heat flow calculations [6]. When systems are stated to have an additional interface resistance, this is implemented by directly scaling this harmonic average. Table SI shows the material properties used in systems shown in Fig. S1 and throughout the main article.

**Material**	κ (W/m/K)	C_V (J/kg/K)	ρ(kg/m^3)	τ ns
White/medium green (homogeneous systems)	220	385	8930	1
White/dark green (metamaterial systems)	40	385	8930	1
Red/light green (metamaterial systems)	400	385	8930	1
Black	100000	0.0001	0.0001	1

Table SI: A table detailing the material parameters for systems is depicted in Fig. S1, where material parameters are thermal conductivity κ, heat capacity C_V, density ρ, and lifetime τ. The term *homogeneous system* refers to the homogeneous disc and bar systems, and its thermal conductivity is the average of the white and red materials used in the *metamaterial systems* (i.e. the hierarchical disc and bar systems).

The choice of the lifetime parameter, τ, for all materials is 10^{-9} s. We have chosen a 'high' value of τ comparable with phonon acoustic modes lifetime to enhance the effects and work with the possible upper limit of τ that could be experimentally used, there is evidence of nanoseconds phonon relaxation times being used for experimental studies used at temperatures around room standard [7].

S3. SUPPORTING RESULTS

In this section, we discuss minor effects such as changing boundary/interface parameters and subtle variations in geometry. This is to provide further support for the main conclusions. These results are to increase the validity/stability of the model to changes in parameters. These changes can result in physically reproducible quantitative differences, but they retain the same qualitative results.

Schematics for supplementary

The system schematics for those in the main text are shown in Fig. S1.

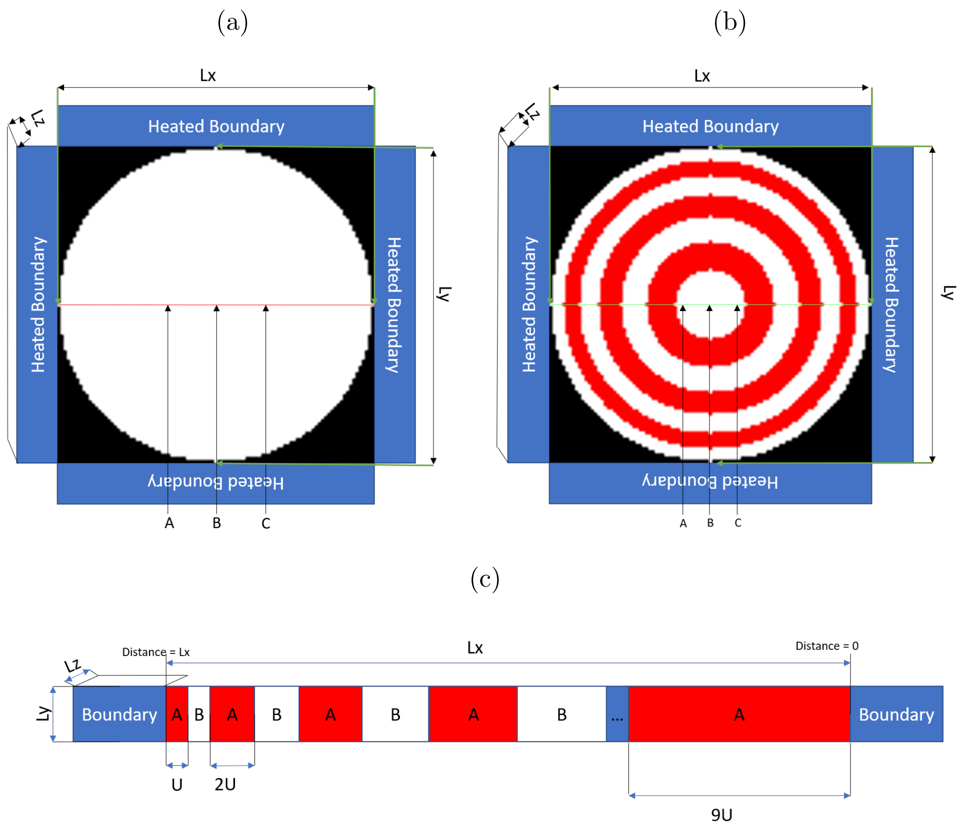

Figure S1: The schematics of the systems. A, B, and C are sampling points. A and C are 0.1 L_x from the centre B on either side. Figure S1a shows the circular system. Figure S1c shows the hierarchical system, $U = L_x/100$. Figure S1b shows concentric rings whose thickness decreases towards the outer edge.

Homogeneous Disc: Larger Dimensions

Fig. S2 shows the Cattaneo behaviour of the homogenous disc with larger system dimensions (Figure S1a, $L_x = 0.1$mm, $L_y = 0.1$mm, $L_z = 1$µm, $\tau = 10^{-9}s$). This has the effect of completely dampening the wave-like behaviour.

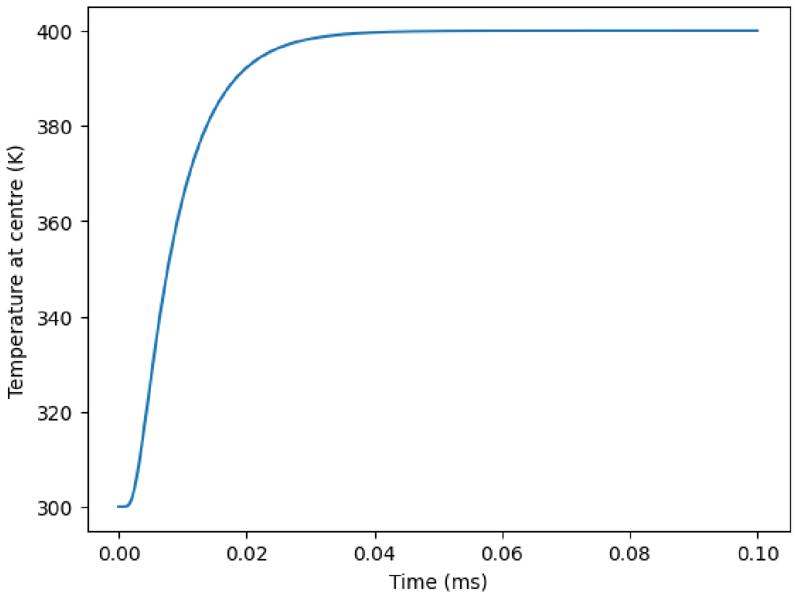

Figure S2: Showing the Cattaneo model behaviour of the centre of the homogenous disc (Figure S1a, $L_x = 0.1$mm, $L_y = 0.1$mm, $L_z = 1$µm), under the condition that the dimensions of the system are much larger compared to the main letter.

Homogeneous Bar: Analytical Solution

We can evaluate Equation S33 for the same material parameters and bar geometry used for Fig. 1(a). We show in Fig. S3 the plot of the analytical solution for temperature for the three points (5, 25, and 45 nm) on the bar. As in the numerical result, we observe the wavefront decaying (with an oscillatory modulation) as has been observed before[8]. We see the result of the forward and backwards propagating waves resulting in a reflection at ≈ 8ns, again agreeing with our numerical simulations.

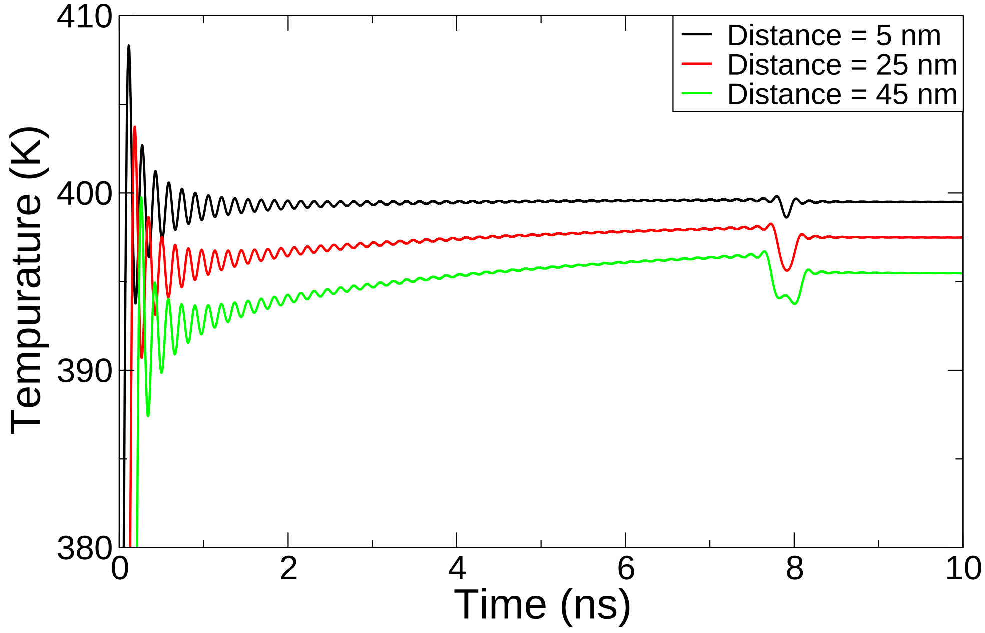

Figure S3: This replicates the data from Fig. 1(a) using the analytical solution. All plots are evaluated including all modes up to n = 50.

Homogeneous Disc: Temperature-Dependent κ and τ Effects

We also investigated the effects of making the thermal conductivity and the phonon relaxation time, temperature dependent, Fig.S4. We did not make density or heat capacity temperature-dependent, as energy conservation poses an additional challenge. We made the thermal conductivity and the phonon relaxation time proportional to the inverse of temperature, with initial values equal to the non-temperature dependence. The thermal conductivity and the phonon relaxation time both go to 0 as the temperature goes to infinity, which is to better show the extreme limit of the behaviour of the system under temperature dependence. It is still clear that the non-Fourier behaviour of the system is shown in Fig.S4. To computationally model Fig.S4, the thermal conductivity and phonon relaxation time of any cell were only changed if the temperature of that cell resulted in a change in the magnitude of the parameter of more than or equal to 10% of its current value.

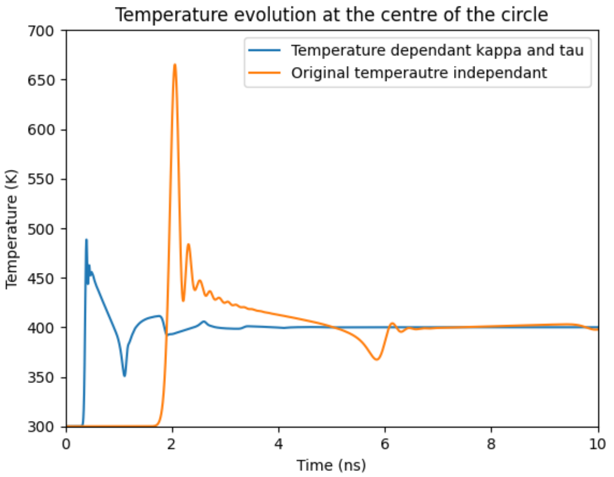

Figure S4: Showing the Cattaneo model behaviour of an initially homogenous disc, under the condition that the thermal conductivity and the phonon relaxation time are temperature-dependent, proportional to the inverse of temperature (Figure S1a, $L_x = 1\mu m$, $L_y = 1\mu m$, $L_z = 1nm$).

Homogeneous Disc: Reduced Relaxation Time

The system shown in Fig.S5 shows that the system under the homogenous disc (Figure S1a) conditions with dimensions, $L_x = 1\mu m$, $L_y = 1\mu m$, $L_z = 1nm$, looses its wave-like behaviour.

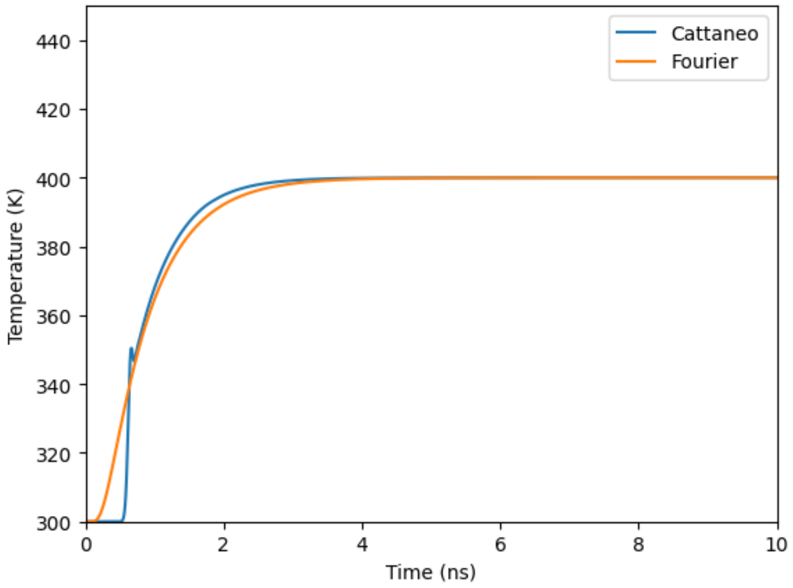

Figure S5: Showing the Cattaneo model behaviour of a homogenous disc (Figure S1a, $L_x = 1\mu m$, $L_y = 1\mu m$, $L_z = 1nm$), under the condition that the τ is a factor of 10 smaller than that shown in the main letter ($\tau = 10^{-9} \rightarrow 10^{-10}s$)

Hierarchical Bar: Reversed Heating Direction

Fig.S6 shows the effect of reversing the grading to that of the system described in the main text. It shows the impact of having the bar heated from the left side boundary of Figure S1c. The initial temperature peaks are much greater than the Hierarchical bar in the main text, but the amplitude appears to decay more quickly. We note that when in steady state, the total heat flux through the bar remains the same regardless of direction. It is only when subjected to the time variance that this asymmetry appears. This is a direct result of the waves having different path lengths when switching the hot and cold ends of the structure.

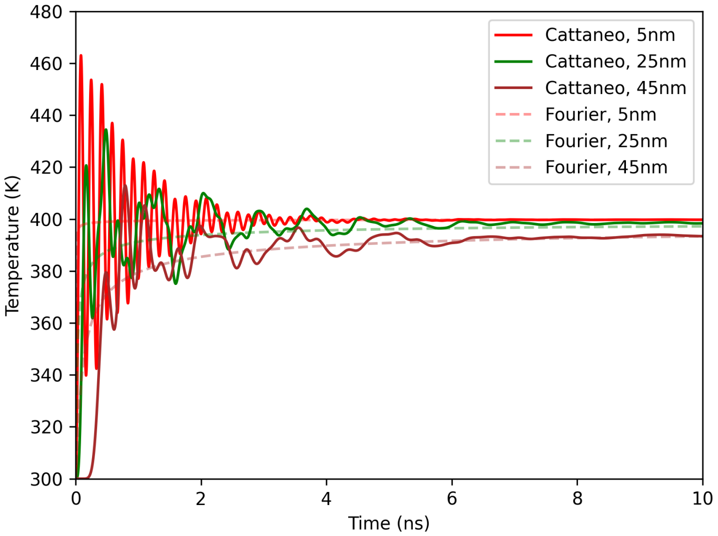

Figure S6: Showing the hierarchical bar system but with the heated boundary (400K) on the other side of the main paper (left side of (c)).

Hierarchical Bar: Conductivity Proportional to Relaxation Time

Fig.S7 shows the effect of linking the thermal conductivity to τ ($\kappa \propto \tau$). The high conductive region has $\tau = 1ns$, and the low conductive material has $\tau = 0.1ns$. As discussed in the main text, the effect of varying the relaxation time in our different media is to increase the amount of reflection at the interfaces, resulting in stronger wave-like characteristics, at the expense of having an increased decay rate due to the smaller relaxation time in the lower conductivity material. Effectively, the inclusion of differing relaxation times for different media leads to further mismatch in the system.

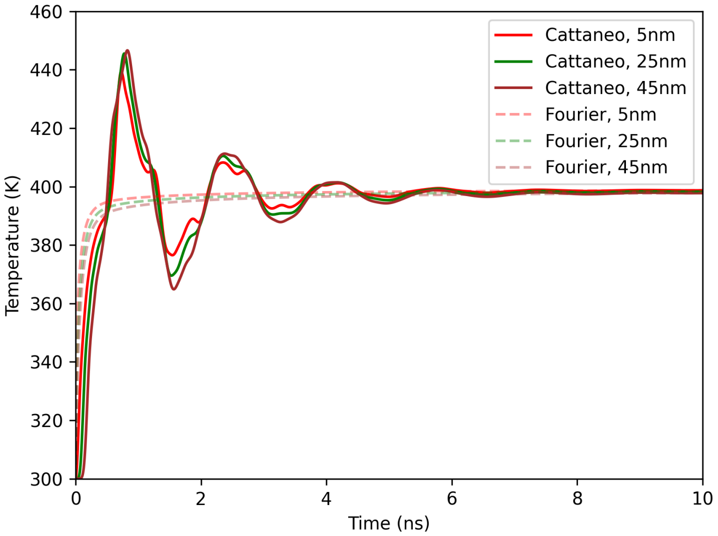

Figure S7: Showing the results, for a varying τ with thermal conductivity in the hierarchical bar ((c)), where the high conductivity material, red, has $\tau = 1ns$, and the low conductive material, white, has $\tau = 0.1ns$.

Hierarchical Disc: Average Temperature Evolution

For hierarchical disc, we present in Fig.S8 the average temperature of the entire system over time (L_x = 1µm, L_y = 1µm and L_z = 1nm), for both Cattaneo and Fourier models. We present this for comparison with the previous result in Fig.S16. These two results show similar behaviour. The hierarchical disc also demonstrates the reflection of the heat wave from the thermal boundaries, which results in an increased number of peaks.

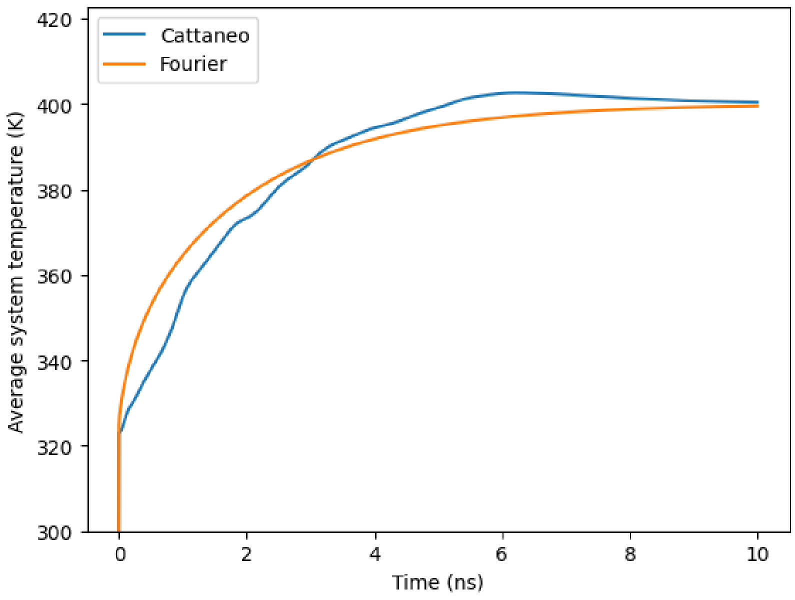

Figure S8: The average temperature of the system over time for the hierarchical disc system (Figure S1b), L_x = 1µm, L_y = 1µm and L_z = 1nm

Periodic Disc: Defect Ring

Here we present the periodic disc (Figure S9a) with dimensions (L_x = 1µm, L_y = 1µm and L_z = 1nm) and with the same material parameters as shown in I. The difference lies in the defect that is introduced, shown in Fig.S9; this defect is a change in the thermal conductivity (20W/m/K) of one of the rings in the system (shown in green). The result is as shown inFigure S9b

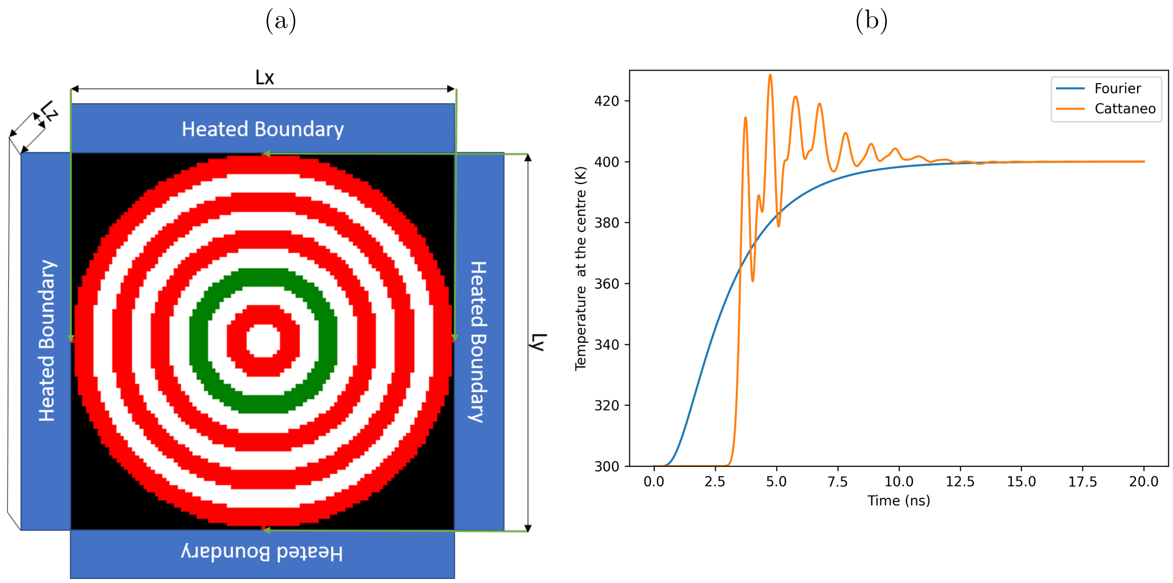

Figure S9: Figure S9a shows the periodic disc system with a defect (shown in green), all parameters apart from green are shown in Table I, the only parameter difference for green is thermal conductivity, which is 20W/m/K.

Hierarchical Disc: Swapped Conductivity Regions

Here we consider the case where the high and low thermal conductivity regions (seen in Figure S1b) are swapped. Fig.S10 shows the temperature of the centre of the system as a function of time. Due to the change in ordering, the result is subtly different. In this system, we see a reduced wave-like effect with increased thermal diffusion, resulting in lower thermal peaks and a shorter signal.

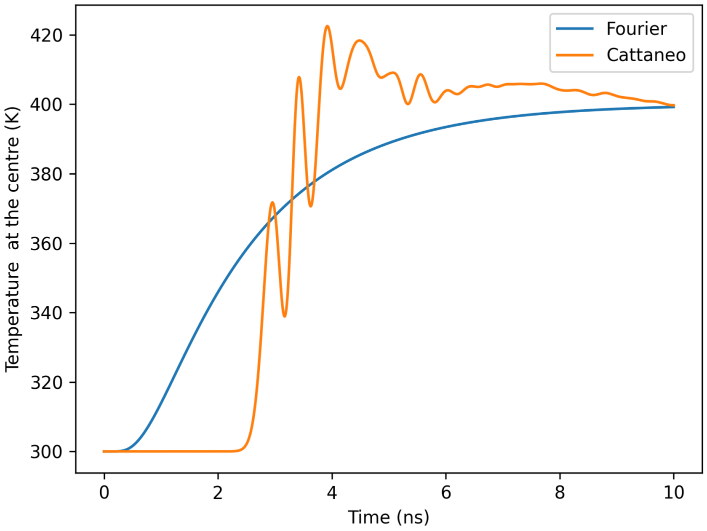

Figure S10: Hierarchical disc system (Figure S1b, $L_x = 1\mu m$, $L_y = 1\mu m$ and $L_z = 1nm$) but red and white material properties have swapped, $L_x = 1\mu m$, $L_y = 1\mu m$ and $L_z = 1nm$. This figure shows the temperature evolution of the Cattaneo model for this system.

Hierarchical Disc: Inverted Hierarchy Grading

In Fig.S11 we present the hierarchical disc but with the radial grading reversed so that rings become thinner toward the centre of the disk. The exact geometry is shown in 11a, which shows the opposite grading of 1b, the results of using the same parameters as the main text are shown in Figure S11b.

(a)

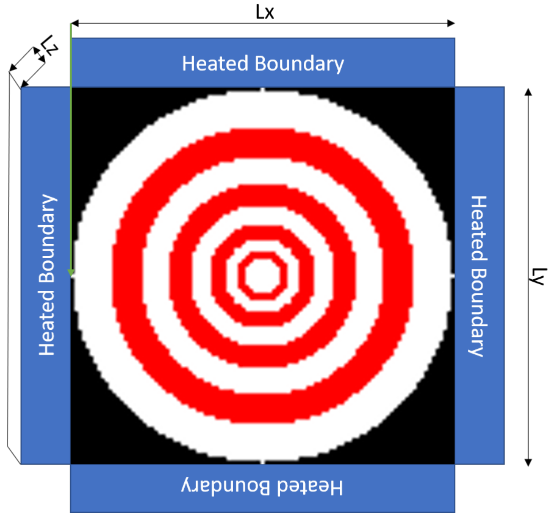

(b)

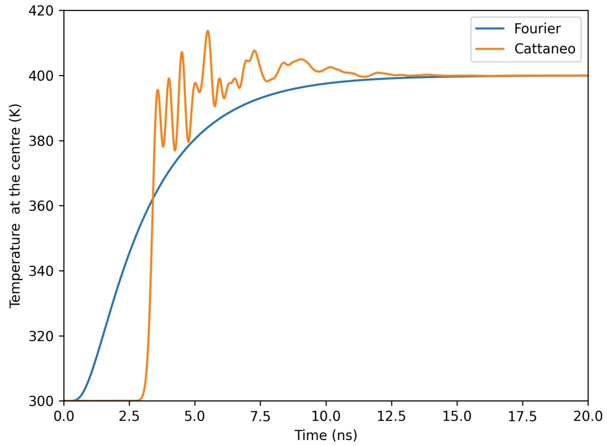

Figure S11: (a) shows the system schematic with thermal property values show in TableSI, $L_x = 1\mu m$, $L_y = 1\mu m$ and $L_z = 1nm$. (b) shows the temperature of the centre of the system in both the Cattaneo and the Fourier models.

Hierarchical Disc: Added Kapitza Resistance

For the hierarchical disc, we consider the effect of interface resistance, Fig.S12. It is known that the interfaces between the two materials have additional Kapitza resistance.

The consequence of the added resistance is that the ratio of the range between the highest and lowest temperature (of the centre) of the added resistance and the added resistance systems is approximately equal to the ratio of the system's total average thermal conductivity. This is at least true for the results shown in Fig.S12. 11210 boundaries are affected by the added resistance. There are a total of 40804 boundaries. Therefore the 0.725% of the boundaries have been changed. This gives a scaling factor of $0.73 + 0.27 \cdot x$ where x is the percentage change in the harmonic average of the interface thermal conductivity.

(a) (b)

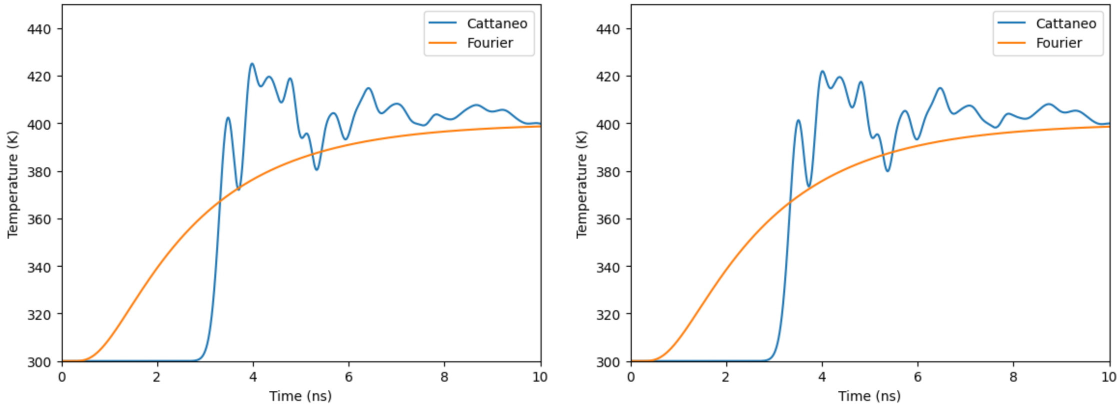

Figure S12: The temperature of the centre of the hierarchical disc system. (a) shows the effect of decreasing the interface thermal conductivity by 1%. (b) shows the effect of decreasing the interface thermal conductivity by 10%.

Homogeneous Bar: High-Boundary-Conductivity Comparison

In Fig.S13, we show an additional set of results for the homogeneous bar system as in the main article. However, in this setup, we modify the boundary (i.e. we set the boundary conductivity to 10^5W/m/K. This conductivity is used for the disc and ring systems boundary. This is to show that the differences between the systems shown in the main paper are not due to the thermal boundary effects nor our choice of the thermal boundary parameter. Effectively, this change shows that the temperature has the same qualitative behaviours, but with slight variation in the magnitude and oscillations due to the greater $\nabla \cdot (\kappa \nabla T)$.

(a) (b)

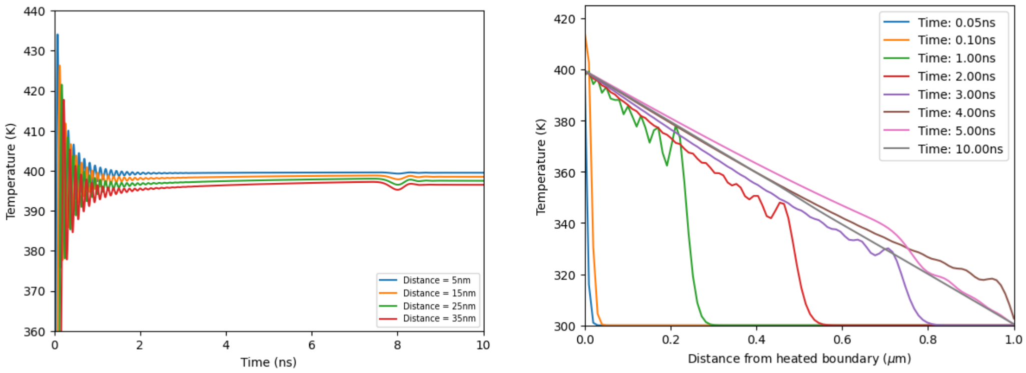

Figure S13: Figures show the same system as the main letter (Figure S1c) but with a thermal boundary conductivity of 10^5W/m/K to be the same as the boundary for the disc and Ring systems.

Hierarchical Bar: High Boundary Conductivity

In Fig.S14, we adjust the thermal conductivity of the boundaries of the system and show the temperature evolution changes as a function of time. Like the homogeneous bar system, the change in thermal conductivity of the boundary shows with slight variation in the magnitude and oscillations due to the greater $\nabla \cdot (\kappa \nabla T)$, but the overall behaviour is the same. In real systems, this contact conductivity is expected to be much lower than in the main letter, but these two cases provide examples of the range of results one could expect.

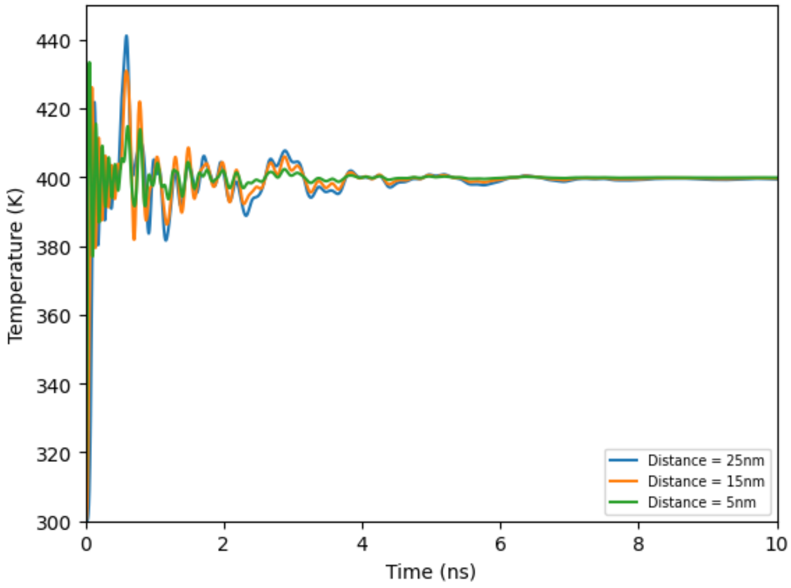

Figure S14: The Hierarchical bar shown in the main letter but with a left and right sink and source thermal conductivity of 10^5W/m/K. This makes them equivalent to the thermal boundaries in the disc systems.

Homogeneous Disc: Zero Heat-Capacity/Density Boundary Region

Fig.S15 shows the homogenous disc system with all parts of the system having thermal conductivity of 220, and the heat capacity, density, and τ of the black region (shown in Figure S1a) are 0. This shows the effect of changing the parameters of the artificial heated boundary to approximate a computational heated boundary around the disc.

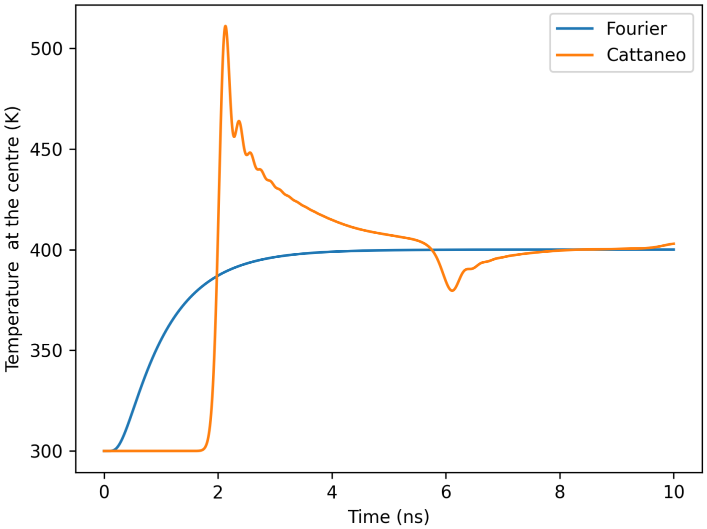

Figure S15: Here we show the homogenous disc system with all parts of the system with thermal conductivity of 220, and the heat capacity, density, and τ of the black region (shown in Figure S1a) is 0.

Homogeneous Disc: Time-Step Convergence of Average Temperature

This section demonstrates that our results are robust against discretisation in the temporal domain. Fig.S16 shows the average spatial temperature across the system in both Cattaneo and Fourier models as the system evolves. It shows that reducing the time step ($10^{-12}s \rightarrow 10^{-13}s$) of the simulation produces an identical average system temperature for both the Cattaneo and Fourier models compared to the longer time step, Fig.S16. This we see as a characterisation of the stability of our computational simulation, showing no change with smaller dt. We also note that when averaging across the entire system, the reduced temperature effect indicates that the greater the area of any thermal measurement, the smaller the detected temperature difference will be than the Fourier result.

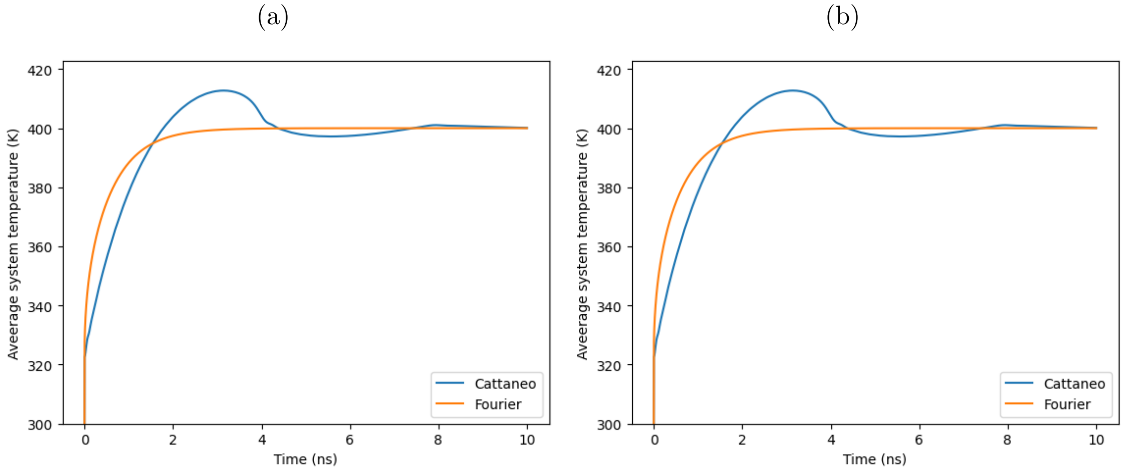

Figure S16: The average temperature of the homogeneous disc system (Fig.S1a, $L_x = 1\text{µm}$, $L_y = 1\text{µm}$, $L_z = 1\text{nm}$), this graph shows the same result for two different simulation time steps($dt = 10^{-12}$, Figure S16a, and 10^{-13}s, Figure S16b, respectively).

REFERENCES

[1] P.G. Klemens. The thermal conductivity of dielectric solids at low temperatures. *Proceedings of the Royal Society of London. Series A. Mathematical and Physical Sciences*, 208(1092):108–133, August 1951.

[2] Joseph Callaway. Model for lattice thermal conductivity at low temperatures. *Physical Review*, 113(4):1046–1051, February 1959.

[3] GyaneshwarP. Srivastava. *The Physics of Phonons.* CRC Press, Boca Raton, August 2022.

[4] R.Kovács. Heat equations beyond fourier: From heat waves to thermal metamaterials. *Physics Reports*, 1048:1–75, 2024.

[5] Charles Kittel. *Introduction to solid state physics.* John Wiley & Sons, Nashville, TN, 8 edition, October 2004.

[6] R.W. Lewis, K.Morgan, and B.A. Schrefler, editors. *Numerical Methods in Heat Transfer*, volume2 of *Wiley Series in Numerical Methods in Engineering.* John Wiley & Sons, Chichester, U.K., August 1983. A Wiley–Interscience publication; selected papers from the 2nd Int. Conf. on Numerical Methods in Thermal Problems, Venice, July 1981.

[7] Albert Beardo, Miquel López-Suárez, LuisAlberto Pérez, Lluc Sendra, MariaIsabel Alonso, Claudio Melis, Javier Bafaluy, Juan Camacho, Luciano Colombo, Riccardo Rurali, FrancescX-avier Alvarez, and JuanSebastián Reparaz. Observation of second sound in a rapidly varying temperature field in ge. *Science Advances*, 7(27):eabg4677, 2021.

[8] A.J. vander Merwe, N.F.J. van Rensburg, and R.H. Sieberhagen. Comparing the dual phase lag, cattaneo-vernotte and fourier heat conduction models using modal analysis. *Applied Mathematics and Computation*, 396:125934, May 2021.
